# Supplementary material for: Circulating Tumor DNA Reflects Uveal Melanoma Responses to Protein Kinase C Inhibition
Source: Cancers (Basel). 2021 Apr 6;13(7):1740. doi: 10.3390/cancers13071740 (PMC8038771; doi:10.3390/cancers13071740)
Supplement: Supplementary file 1 [file cancers-13-01740-s001.pdf]

cancers

MDPI

Supplementary Materials: Circulating Tumor DNA Reflects Uveal Melanoma Responses to Protein Kinase C Inhibition

John J. Park, Russell J. Diefenbach, Natalie Byrne, Georgina V. Long, Richard A. Scolyer, Elin S. Gray, Matteo S. Carlino and Helen Rizos

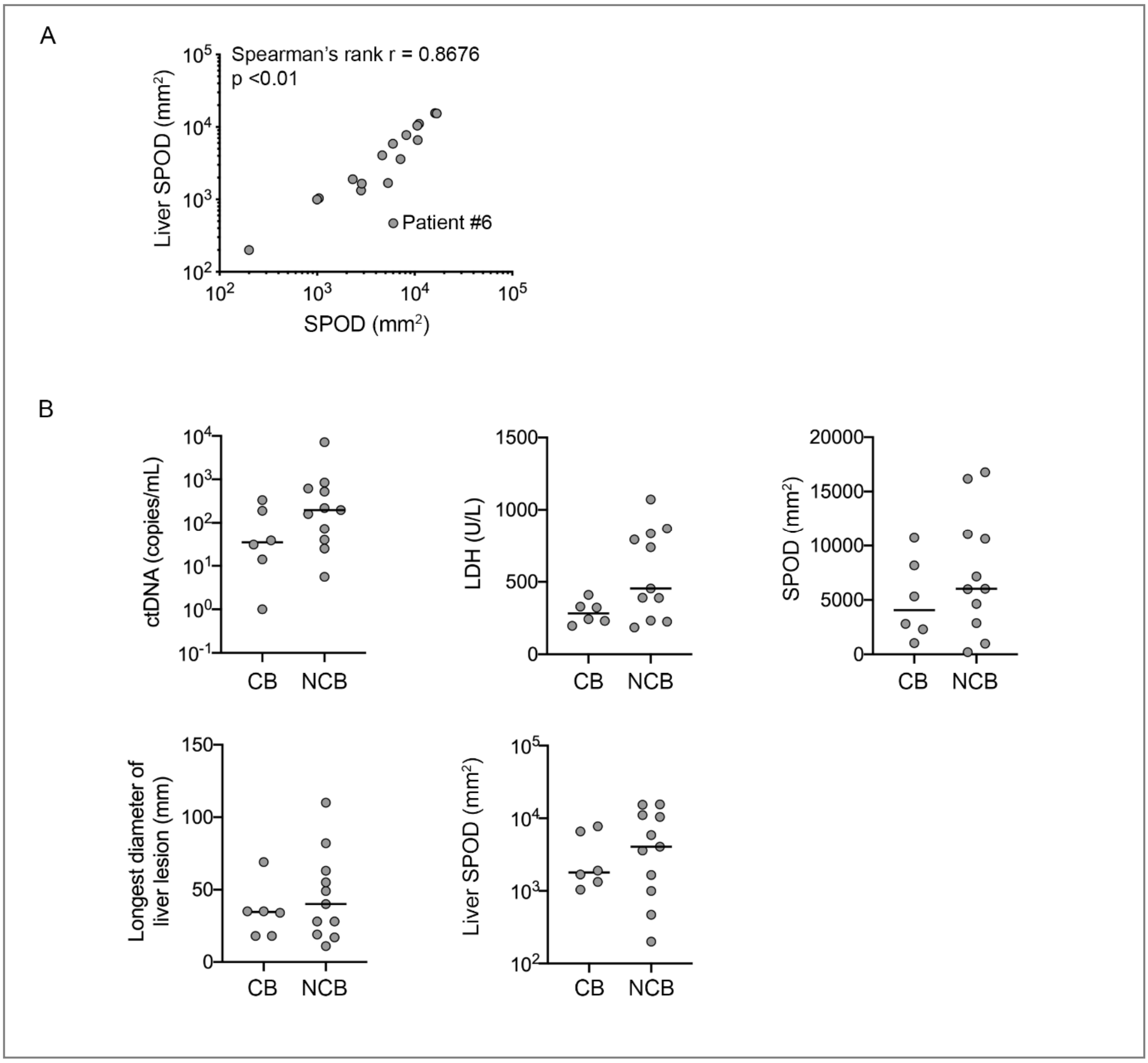

Figure S1. Relationship between PKC inhibitor-based response and ctDNA levels, LDH and disease burden. (**A**) Spearman's rank correlation between SPOD (mm^2) and liver SPOD (mm^2) ($p < 0.01$). Patient #6 had multiple disease sites, and a relatively low level of liver disease. (**B**) Baseline ctDNA levels, LDH, SPOD, longest liver lesion, and liver SPOD were consistently, but not significantly lower, in clinical benefit (CB) (PR and SD > 6 months) compared to no clinical benefit (NCB) (PD and SD < 6 months) UM patients. Graphs show ctDNA+1. Kruskal-Wallis test with Dunn's multiple comparison test was used to compare untransformed ctDNA copies in clinical benefit group and no clinical benefit group.

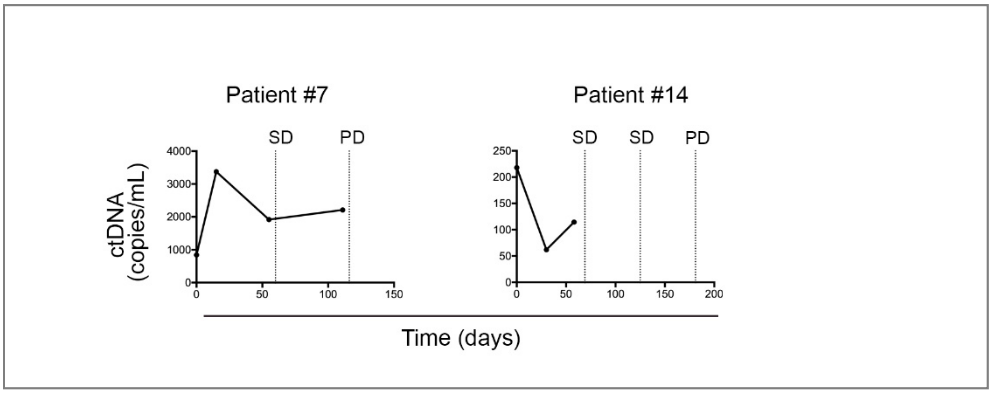

Figure S2. Longitudinal monitoring of ctDNA in patients treated with PKCi in metastatic UM. ctDNA levels were collected longitudinally during treatment and correlated to CT imaging during baseline, whilst on treatment and on progression. Longitudinal ctDNA monitoring is shown for no clinical benefit patients #7 and #14. SD—stable disease, PD—progressive disease.

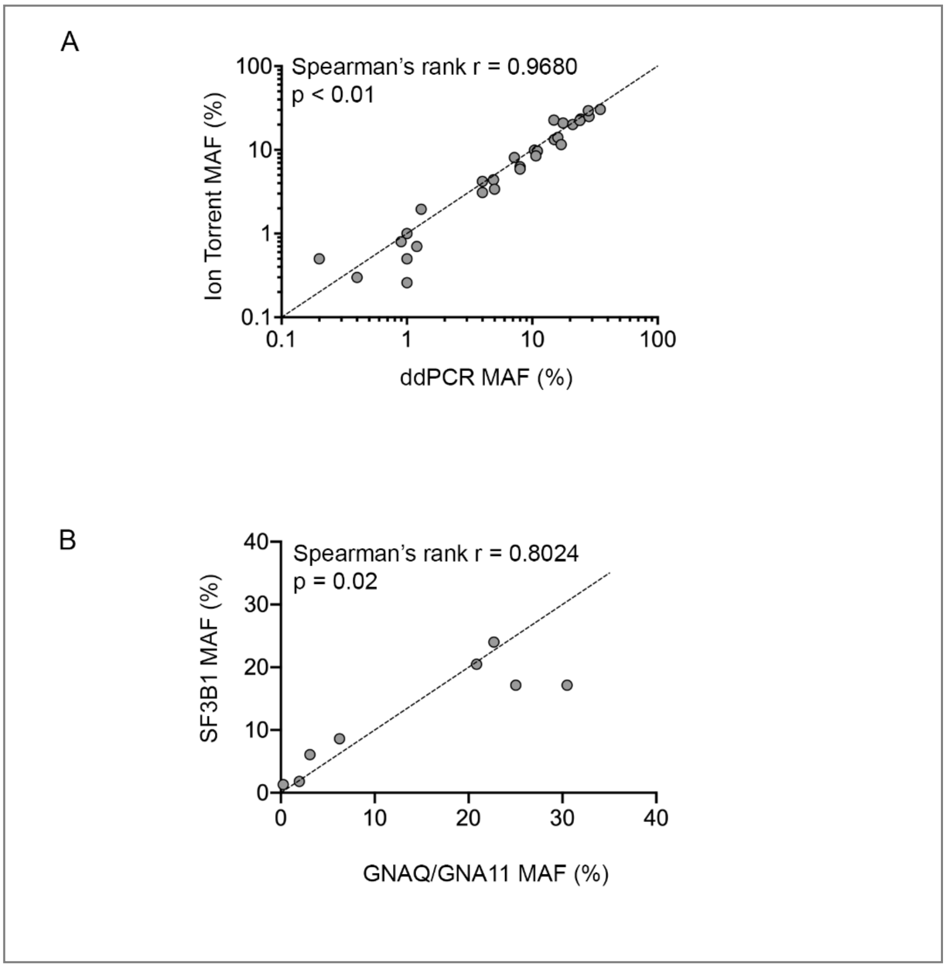

Figure S3. Significant correlation in mutant allele frequency determined by ddPCR and targeted Ion Torrent NGS. (**A**) Spearman's rank correlation between mutant allele frequency (MAF) of ddPCR and Ion Torrent NGS. Correlation was performed on untransformed mutant allele frequency values. (**B**) Spearman's rank correlation between mutant allele frequency (MAF) of Ion Torrent NGS comparing SF3B1 mutation and GNAQ/GNA11. Dashed lines are y = x.

Table S1. Patient and response to therapy details.

Patient ID	Age	Sex	ECOG PS	Mutation	Best Response	SPOD (mm^2)	Liver SPOD (mm^2)	Longest Liver Lesion (mm)	Baseline LDH (U/L)	Baseline ctDNA (Copies/mL)	Sites of Disease	Treatment	Dose of PKCi	Primary Tumour Type	PFS (Months)
1	56	M	0	GNA11^{Q209L}	SD ≥ 6 mths	8178	7741	34	323	37.5	Liver, bowel	PKCi	300mg OD	Choroidal	7.4
2	71	M	0	GNA11^{Q209L}	PR	2316	1896	35	231	30	Liver, retroperitoneal	PKCi	300mg BD	Unknown	3.7
3	55	M	0	GNA11^{Q209L}	SD ≥ 6 mths	2814	1326	18	243	13	Liver, lung, bone, omentum	PKCi	300mg OD	Choroidal	9.6
4	69	F	0	GNAQ^{R183H}	SD ≥ 6 mths	10726	6604	69	411	187.5	Liver, lung, LN	PKCi	400mg BD	Iris	9.3
5	47	M	0	GNA11^{Q209L}	SD ≥ 6 mths	1041	1041	18	197	0	Liver	PKCi	300mg BD	Unknown	13.1
6	51	M	0	GNAQ^{Q209P}	PD	6022	469	17	1072	7172	Liver, lung, adrenal, bone, LN	PKCi	500mg OD	Choroidal	1.9
7	45	M	0	GNA11^{Q209L}	SD < 6 mths	11063	11063	63	793	842	Liver, bone	PKCi	300mg OD	Choroidal	3.6
8	54	F	0	GNAQ^{Q209P}	PD	5986	5881	40	390	157.7	Liver, lung	PKCi	300mg BD	Unknown	1.7
9	57	F	0	GNA11^{Q209L}	SD < 6 mths	200	200	11	186	24	Liver	PKCi	300mg BD	Unknown	3.7
10	67	F	0	CYSLTR2^{L129Q}	PD	4635	4072	49	233	196.2	Liver, bone, spleen	PKCi	300mg BD	Choroidal	1.9
11	52	F	0	GNA11^{Q209L}	SD < 6 mths	16175	15525	55	739	614	Liver, lung	PKCi	400mg BD	Choroidal	3.8
12	67	F	1	GNA11^{Q209L}	PD	2871	1653	19	389	39	Liver, lung, bone, LN	PKCi + HDM2i	300mg OD	Choroidal	1.7
13	67	F	0	GNAQ^{Q209P}	SD < 6 mths	997	997	28	225	15.4	Liver	PKCi + HDM2i	100mg BD	Choroidal	5.4
14	55	M	1	GNAQ^{Q209P}	SD < 6 mths	16782	15366	82	454	218	Liver, lung, LN	PKCi + HDM2i	200mg BD	Choroidal	3.8
15	73	M	1	GNAQ^{Q209P}	SD < 6 mths	7154	3595	28	835	521	Liver, lung, LN, spleen	PKCi + HDM2i	300mg BD	Choroidal	3.8
16	55	M	1	GNAQ^{Q209P}	PR	5330	1682	35	329	333	Liver, lung bone, LN, pancreas	PKCi + HDM2i	300mg BD	Choroidal	3.7
17	67	M	0	GNAQ^{R183Q}	SD < 6 mths	10628	10460	110	869	71	Liver, spleen, bone	PKCi + HDM2i	400mg BD [a]	Choroidal	3.9

Abbreviations: LDH, lactate dehydrogenase; PR, partial response; SD, stable disease; PD, progressive disease; SPOD, sum of the product of bi-dimensional diameters; PFS, progression free survival; LN, lymph node; U/L, units/litre; PKCi, protein kinase C inhibitor (LXS196); HDM2i, human double minute 2 inhibitor (HDM201); ECOG PS, Eastern Cooperative Oncology Group performance status; mths, months; OD, once daily; BD, twice daily. [a]Patient #17 had a 3 day does interruption during the PRE-EDT sample period and was on treatment at the time of EDT sample collection.
